# Conducting Patient-Oriented Research in Pediatric Populations: A Narrative Review

**DOI:** 10.3390/children11101266

**Published:** 2024-10-19

**Authors:** Alan P. Cooper, Linda Nguyen, Oluwapolola Irelewuyi, Steven P. Miller

**Affiliations:** 1 Department of Paediatrics, The Hospital for Sick Children, Toronto, ON M5G 1X8, Canada; irelewo@mcmaster.ca (O.I.); steven.miller@cw.bc.ca (S.P.M.); 2 Neurosciences & Mental Health, SickKids Research Institute, Toronto, ON M5G 0A4, Canada; 3 Faculty of Social Work, University of Calgary, Calgary, AB T2N 1N4, Canada; linda.nguyen1@ucalgary.ca; 4 Faculty of Medicine and Health Sciences, McGill University, Montreal, QC H4A 3J5, Canada; 5 CanChild Centre for Childhood Disability Research, McMaster University, Hamilton, ON L8S 1C7, Canada; 6 Faculty of Health Sciences, McMaster University, Hamilton, ON L8S 4L8, Canada; 7 Department of Pediatrics, University of British Columbia, Vancouver, BC V6T 1Z4, Canada; 8 BC Children’s Hospital Research Institute, Vancouver, BC V5Z 4H4, Canada

**Keywords:** patient-oriented research, patient and public involvement, research engagement, patient-partner, person with lived experience, diversity and inclusion in research, Canadian Institutes of Health Research (CIHR) pediatric research, and childhood disability research

## Abstract

It has become increasingly common for researchers to partner with patients as members of the research team and collaborate to use their lived experiences to shape research priorities, interventions, dissemination, and more. The patient-oriented research (POR) model has been adopted by both adult and pediatric health researchers. This cultural change to conducting pediatric health research brings with it new methodologies, tools, challenges, and benefits. In this review, we aim to provide guidance on how to conduct POR for pediatric populations using examples from the literature. We describe considerations for engagement before the project begins, for engagement across the research cycle, and for measurement and evaluation. We aim to show that conducting POR is feasible, beneficial, and that many common challenges and barriers can be overcome with preparation and usage of specific tools.

## 1. Introduction

The goal of health research is to produce new evidence, treatments, and interventions to improve the outcomes of patients. Paradoxically, researchers and clinicians have traditionally determined health research agendas and priorities without patient input. The absence of patient perspectives in determining research priorities and design may result in studies and healthcare practices that are disconnected from the patient’s needs. Furthermore, conducting health research without the input of patients and end users can contribute to research waste [1].

To address these shortcomings, over the last few decades, research funders such as the National Institutes for Health Research (NIHR, London, UK), National Institutes of Health (NIH, Bethesda, USA), and the Canadian Institutes of Health Research (CIHR, Ottawa, Canada) have begun to shift toward a more inclusive research model [2,3,4]. Various terms are used to describe this approach to research, including patient and public involvement in research, patient-centered outcomes research, patient-involved research, patient engagement in research, family-oriented research, and patient-oriented research. Integrated knowledge translation is another collaborative approach that encourages the engagement of knowledge users (e.g., patients, clinicians, policymakers) throughout the research process, and knowledge translation during the project and at the end of the project [5]. In Canada, this research approach is termed patient-oriented research (POR), which is the term we use in this review. CIHR defines POR as “continuum of research that engages patients and the public as partners to focus on patient-identified priorities and improve patient outcomes” [6]. POR is underpinned by moral and political principles [7,8]. Morally, patients have a right to be involved in research that impacts them or those with similar conditions [9,10]. Politically, engaging the public in taxpayer-funded research encourages research that will meet the needs and priorities of the public.

Various terms exist to describe the partnership role that a patient can have in research, including a person with lived experience, youth research partner, parent research partner, consumer, and patient-partner. For this review, we will use the term patient-partner, which is the current terminology used by CIHR. A patient-partner can refer to an individual with lived and living experience as a patient or with the healthcare system, as well as their family and caregivers [6]. In the context of POR for pediatric populations, a patient-partner could be a child or a youth who is a patient, a former pediatric patient, or a sibling, parent, or caregiver of a child or youth patient. A patient-partner is an active member of the research team, and they can contribute during all stages of the project, including priority setting, study conduct, and knowledge mobilization. Notably, the role of a patient-partner is distinct from the role of a research participant, who is someone who consents to take part in a study and provides information to the research team as a source of data.

POR funding initiatives from the NIHR, NIH, and CIHR have allowed research with patient-partners to flourish. POR methodology has primarily been applied to clinical research, which is the focus of this review. However, POR is also being applied to a range of other types of health and medical research, including implementation research, quality improvement processes, secondary research, and pre-clinical research [11,12,13,14]. We are now in a better position to describe how to engage patient-partners in research and identify benefits, challenges, and strategies to overcome these challenges.

Engaging patient-partners in research is associated with benefits for the project, the researcher, and the patient-partner. At the project-level, benefits include the identification of research priorities relevant to the patient, interventions that are more acceptable to the patient, and dissemination of products that reach broad audiences and are directed at lay audiences [15]. Researchers benefit by approaching their research from a patient perspective and by learning new skills, such as how to integrate different viewpoints into research and how to negotiate with diverse team members [16]. Patient-partners may benefit from engaging as partners in research by gaining knowledge, building new networks, feeling empowered, and helping others [17,18].

Conducting POR is also associated with a range of challenges [19]. Engaging patient-partners can increase the time and resources required to complete a project, and it takes time and experience for patient-partners and researchers to learn how to conduct POR [15]. Patient-partners have reported that they can lack clarity on their role, are confronted with a power imbalance, and receive too little communication from researchers, leading to tokenistic engagement [20]. Similar challenges have been reported in the pediatric population, where children and youth may lack confidence to engage with or feel intimidated by working with unfamiliar people [21]. Researchers have reported challenges identifying and recruiting patient-partners [22] and lack clarity on how and when to engage patient-partners in research activities [17].

In this review, we provide guidance on how to conduct POR for pediatric populations. We provide considerations for conducting pre-study activities and describe how to engage patient-partners throughout the research cycle. To illustrate the scope of work involved, we provide examples from the literature that provide a thorough description of the participatory approach used by researchers at each research stage. While it was a challenge to find thorough descriptions of each stage in the pediatric literature, we also shared our experiences in POR and consulted with patient-partners in our networks. Lastly, we provide guidance on how to report and evaluate POR and engagement activities. We aim to show that conducting POR in pediatric populations is feasible, beneficial, and that many common challenges and barriers can be overcome with preparation and usage of specific tools.

## 2. Search Methods Statement

For this narrative review, we conducted ad hoc searches on PubMed and Google between September 2023 and August 2024. Search terms included patient-oriented research, patient and public involvement, patient-partner, person with lived experience, diversity and inclusion in research, pediatric research, and childhood research. We also sought primary literature cited in systematic, scoping, and rapid reviews conducted in patient-oriented research for pediatric and adult populations [16,21,23,24]. We limited our search to literature published in English.

In addition to reviewing literature, we consulted with three patient-partners (parents) in our networks to understand their experiences being engaged in research, and to understand their perspectives on measuring the benefits and impacts of engagement in research.

## 3. How to Begin POR: Pre-Study Activities

In the next section, we provide an overview of the preliminary steps involved with POR: building respectful relationships, recruiting patient-partners, and administering a POR project. Each of these steps requires time: time to build relationships, time to recruit patient-partners, and time to establish administrative processes and documents. Devoting time to these pre-study activities can be a challenge if the research team is pressured to initiate the project. We hope that the strategies listed below prepare researchers to conduct these activities efficiently and provide practical solutions for relationship building, patient-partner recruitment, and administration. A summary of suggestions for how to involve patient-partners throughout the research cycle is provided in Table 1.

### 3.1. Building Respectful Relationships

At the core of POR is a relationship between a researcher and a patient-partner. Successful engagement requires that the relationships between individuals be respectful, safe, and comfortable. POR involves making shared decisions about research. Traditionally, researchers have held the power to make these decisions alone. However, maintaining an environment where researchers continue to hold all the power can lead to patient-partners feeling unheard and undervalued [20,25]. Opportunities can be provided throughout the project where partners and researchers can reflect on partnership experiences, including what is working well and how things can be improved, to ensure that all partners feel included in the research process.

In order to have a healthy relationship with patient-partners, researchers need to recognize this power imbalance and revise how decisions are made. At the beginning and throughout the project, the purpose of inviting patient-partners to projects should be clearly communicated. The knowledge being shared by patient-partners and researchers should be valued in how this knowledge can inform and guide the research project [26]. Clarifying the roles of patient-partners, such as whether they have shared decision-making responsibilities, can help to reduce the power imbalance. It is also important to effectively use interpersonal skills such as active listening, focusing on building trust, being open-minded, being empathetic, and being aware of body language. When partnering with youth, there should also be opportunities for team building and social activities, such as attending day trips or workshops together, sending e-gift cards to order meals during virtual meetings, or sending care packages [26].

Furthermore, team training in conflict resolution and cultural sensitivity can resolve disagreements when they arise and guide individuals on how to work in a diverse group. Important questions should be reflected on with the team, including how to incorporate feedback, ask thoughtful questions about the lived experiences of patient-partners, how to create more inclusive spaces with patient-partners, and how to share the power and privilege with patient-partners [20]. Lastly, researchers can orient patient-partners by explaining the constraints and pressures that researchers operate under, thereby allowing patient-partners to better understand the research context and align their expectations for the pace and process of research.

Establishing comfortable relationships with patient-partners is a critical, early step in POR and underpins subsequent engagement activities; time should be reserved to build rapport before the project begins to ensure that the strategies listed above are considered.

### 3.2. Recruiting Patient-Partners

Recruiting patient-partners can be challenging, especially for researchers who are new to POR [22]. Here, we describe some ways in which patient-partners can be recruited.

A common place to begin is by connecting with individuals who were involved in a previous study as participants and asking if they would be interested in becoming patient-partners in a future study. A researcher can put a general call out to participants at the end of the study to see if they would be interested in partnering on a future project, or a researcher could selectively invite participants with whom they had a good rapport. Notably, in this approach, it is important to be aware of the power dynamics that existed while the individual was a study participant, as this prior relationship could influence the power dynamics if they join subsequent work as a patient-partner.

As the POR movement grows, there are more opportunities for patient-partners and researchers to cross paths and network. Patient-partners now commonly attend conferences, training sessions, and fundraising events. They may also participate in peer review sessions and sit on committees. These are all places for researchers to develop relationships with patient-partners who might share similar research interests. A strength of recruiting from the above options is that both parties can begin to build rapport, assess compatibility, and identify mutual interests in research.

Patient-partners can also be recruited by using an online matching tool. Some organizations have developed matching tools whereby researchers list available patient-partner opportunities, detailing desired skills and experiences, while patient-partners list their skills, interests, and areas of expertise [27,28,29]. Staff members then review applicants, assess suitability, and facilitate a match where appropriate. While matching tools are resource intensive, this method may make the most sense when there are a significant number of matches to be made.

Making an effort to recruit a diverse group of patient-partners is crucial, as this can result in a more inclusive project and intervention. Patient-partner populations tend not to reflect the diversity of the general population, rather, they are often overrepresented by white, middle-class women, and lack diversity [30,31,32]. The recruitment methods mentioned above are often biased toward patients who can attend conferences or participate in studies. Study participant populations have traditionally lacked diversity [33], so if drawing patient-partners from a pool of study participants with a homogenous background, it is likely that the group of patient-partners will not be diverse. This lack of diversity has led to active calls to diversify patient-partner populations using purposive recruitment techniques, such as connecting with community-based organizations that work with diverse populations [34]. Recruiting youth patient-partners also requires purposive recruitment strategies, such as recruiting from schools and community organizations and using social media and websites [21].

### 3.3. Clarifying Roles and Establishing Patient-Partner Committees

Once patient-partners have been recruited to join a project, it is important to clarify the roles of the research team members and patient-partners. Here, we will provide guidance on how to orient research staff before they begin to work with patient-partners. We will also provide an overview of three tools (terms of reference, patient-partner agreement, and the Involvement Matrix) that can be used to guide the formation of a patient-partner committee and support the establishment of roles that patient-partners will have throughout the research project.

Prior to commencing work with patient-partners, researchers (e.g., primary investigators and research staff) should reflect on the skills they will require to manage a POR project. For example, it is beneficial for research staff to have sound administrative and project management skills to ensure that patient-partners possess a clear understanding of their collective tasks. It is also important for research staff to receive training in POR [35,36,37], have peer support, and account for the time-intensive nature of POR.

Often, patient-partners will consolidate to form a patient-partner committee, also known as Patient and Family Advisory Councils [38]. The committee works with the researchers for the duration of a project. It is suggested to use terms of reference to outline how the committee will operate. The terms of reference should include elements, such as the purpose of the committee, background information about the project, membership, roles and responsibilities, and meeting schedules [39], and be agreed upon by patient-partners.

Next, it is critical to define, clarify, and negotiate the role of each patient-partner by developing individual agreements or contracts. This can be conducted by holding one-on-one meetings to understand their interests in the project. These agreements include many of the same elements contained in the terms of reference (meeting schedule, medium of engagement, time commitments), but are tailored to the individual patient-partner. The patient-partner agreement should also describe how they will be compensated. Research organizations now recognize the importance of compensating patient-partners for their contributions, and there are several compensation guidelines that offer detailed guidance [40,41]. Most importantly, a patient-partner agreement should detail their role in the project.

**Figure 1:**
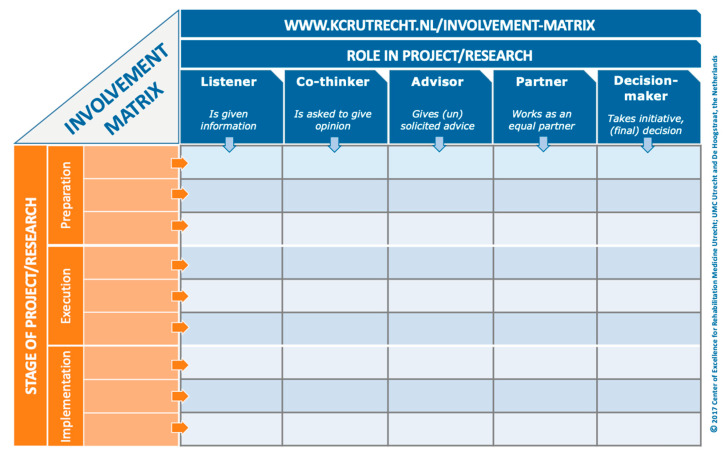
Involvement Matrix [7], www.kcrutrecht.nl/involvement-matrix (accessed on 15 August 2024). © Center of Excellence for Rehabilitation Medicine Utrecht, used with permission.

When a patient-partner is determining their role in a project, they can either be involved in every stage of the research process, or in select stages (e.g., protocol development, recruitment, and data collection) based on their interests and availability. Moreover, patient-partners can be engaged at different levels for each stage. The Involvement Matrix is a conversation tool that can be used to develop a patient-partner’s role in a project (Figure 1) [7]. The Involvement Matrix lists research stages vertically, and the level of engagement horizontally. Through conversation, the patient-partner can determine the role they were serving at each research stage. We recommend that researchers take the time to use a tool, such as the Involvement Matrix, when onboarding each patient-partner to a project in order to provide them with a detailed understanding of their role. This can be completed with the patient-partner at the same time as the individual patient-partner agreement. These documents can be reviewed and revised every 6–12 months, as the project evolves, and new opportunities arise. Reviewing these documents not only ensures that researchers and patient-partners are clear about their roles, but also encourages adherence to the agreement. Having a detailed description of the patient-partner’s role in a project from the beginning will facilitate other aspects of the project, such as measurement and evaluation of engagement, at the end of the project.

## 4. Engaging Across the Research Cycle

Below, we will describe how to engage patient-partners in the major phases of the research cycle: study preparation (setting research priorities, developing a study protocol and intervention), study execution (participant recruitment, data collection, data analysis and interpretation), and knowledge mobilization (also known as knowledge translation). We will describe the benefits and challenges associated with engagement at each stage. Where possible, we will use examples from the literature to provide a thorough description of the participatory approach used by the research team, so the reader has a sense of the process of engagement and the scope of work involved.

### 4.1. Study Preparation Phase

#### 4.1.1. Setting Research Priorities

Research priorities are typically determined by researchers and academic clinicians based on gaps they observe in the literature, or needs they observe in patients in their clinics. Unfortunately, it has been uncommon for patients to input on research priorities. However, it is particularly important to get patient input at this early stage because it determines the aims of the project and sets the tone for subsequent engagement. Moreover, engaging diverse audiences in research priority-setting exercises can identify under-researched questions that might not have been identified without patient-partners being at the table.

Priority-setting exercises can engage patient-partners in activities, such as focus groups, surveys, workshops, and interviews. These exercises can be used to inform priorities for a specific grant and laboratory or for institutional initiatives. Many researchers and clinicians, however, do not have training in conducting broad priority setting and strategic planning initiatives that engage patient-partners and the public. The James Lind Alliance (JLA) has developed rigorous methods to prioritize research questions from patients, caregivers, researchers, and clinicians in activities they term Priority Setting Partnerships (PSPs) [7,42]. Here, we will describe a PSP led by the JLA to highlight how research priorities can be co-developed.

In 2011, the JLA began to work with a group of researchers, patients, and caregivers in the United Kingdom to conduct a PSP that would establish research priorities for people affected by preterm birth [43]. First, a broad list of unanswered research questions was generated from systematic reviews and a publicly accessible survey. There were 386 respondents to the survey, including 247 people affected by preterm birth, either patients or caregivers. After analyzing the questions identified through the systematic review and public survey responses, the group identified 104 relevant research questions. Next, the 104 questions were prioritized using a public vote (507 voters, including 286 people affected by preterm birth) and subsequent workshops that included people affected by preterm birth and healthcare professionals. This process took approximately 2 years to complete. In the end, a list of the top 10 research priorities was generated; patient-partners, researchers, and clinicians agreed on the top priority, but the rankings of other research priorities varied by group.

Co-identifying research priorities, including with children and youth, requires time, resources, and skills. There can also be challenges with engaging with children and youth in priority-setting exercises due to power imbalances and increased workload [44]. Researchers often face time pressures and deadlines to write grant applications and might not have the funds to pay workshop facilitators or to compensate patient-partners for participating in these exercises. JLA-PSPs are particularly thorough and resource-intensive, and thus not always a practical option.

Fortunately, each JLA-PSP is published open access. To date, the JLA has conducted or guided over 200 research prioritization exercises, including several directed at pediatric populations [44,45]. A research group can accelerate research prioritization by using relevant, published PSPs as a starting point when working with their own patient-partners. Researchers can also apply for specific grants to fund prioritization exercises or use funds from a previous grant.

Engaging patient-partners in research prioritization can be beneficial to a project and research team as it ensures that the subsequent research is relevant to patients and sets the tone for engagement in subsequent activities.

#### 4.1.2. Developing a Research Protocol and Intervention

After research priorities are determined, best practice POR suggests the research team go on to co-develop the grant proposal, study protocol, and study intervention with patient-partners. Here, we will focus on how patient-partners can be involved in developing the study protocol post-funding award and the intervention.

Patient-partners should be involved in identifying the research questions, specifying the research aims, and informing the study design. They should also be involved in selecting meaningful outcome measures, proposing appropriate questionnaires, and determining inclusion and exclusion criteria for participants. This is also a stage when patient-partners can contribute to the design of the recruitment strategy and recruitment flyers and co-develop consent forms to ensure the language is appropriate. A common barrier in this phase is that patient-partners might be unfamiliar with the terminology and contents of a study protocol. We recommend budgeting sufficient time to orient patient-partners to the contents and language used in protocols and recognize that some may prefer to engage in these activities either at patient-partner committee meetings or individually.

Next, we consider the involvement of patient-partners in developing an intervention, using the example of a mobile health application. In 2017, Castensøe-Seidenfaden et al. set out to develop a mobile health app to support youth self-managing type 1 diabetes [46]. The research team co-developed the app with youth aged 14–26 years with type 1 diabetes, their parents, and healthcare providers. The multi-stage co-development process included workshops to develop content, prototyping the app, testing the app, then conducting a feasibility study. The co-development of the app was comprehensive and took two years. Although the development was thorough, not all suggestions could be incorporated due to time and financial constraints. The authors also noted that feedback shared was sometimes conflicting; however, they made a point to acknowledge and respect the variation in input. While developing a protocol and intervention with patient-partners is resource-intensive, it can be a worthwhile upfront investment to ensure that the project and intervention are centered around the end user.

### 4.2. Study Execution Phase

#### 4.2.1. Participant Recruitment

Traditionally, participant recruitment in health research is conducted by project staff within healthcare and academic institutions. Identifying and recruiting participants is a well-described challenge that can slow project progress. Further, it can be difficult to recruit a diverse and representative participant sample. Patient-partners can facilitate recruitment by reaching potential participants that researchers cannot, either through their patient-only social media groups or in community settings [47,48].

Patient-partners can become directly involved in recruitment in multiple ways. For example, Rosen-Reynoso et al. designed a program to help youth with disabilities prepare for the transition from secondary to post-secondary education [49]. The study team recruited participants from community settings in order to increase diversity in their sample. Among their recruitment strategies, the study team hired 6 youth aged 16–21 years to promote the study and identify potential participants in the community over summer break. The youth distributed flyers at social events (e.g., parties, clubs, concerts), libraries, summer job fairs, and to their teachers and principals. Although this example does not indicate the relative success rate of this approach, it does describe steps that researchers can take to engage patient-partners with in-person recruitment strategies.

Patient-partners often report being eager to assist with recruitment directly, with the hope that their assistance will help patients in the future [48]. Patient-partners also stress the importance of being authentically engaged throughout all stages of the research project; only being asked to support recruitment can feel transactional from a patient-partner perspective [50].

#### 4.2.2. Data Collection

Data collection has typically been conducted exclusively by research staff; however, an increasing number of studies report involving patient-partners in collecting data. Most commonly, patient-partners have been involved in conducting qualitative interviews and focus groups [51]. Some participants might be more comfortable being interviewed by a patient-partner than a researcher, leading to the collection of data that might not otherwise be collected. Engaging patient-partners in data collection requires training on how to conduct and facilitate activities such as interviews and focus groups. Patient-partners will also need to be oriented to ethical issues associated with data collection, such as maintaining confidentiality, avoiding bias, and collecting data accurately [52]. Patient-partners should also receive support in the instance that participants recount stories that trigger traumatic memories from the patient-partner’s lived experience.

#### 4.2.3. Data Analysis and Interpretation

Traditionally, data analysis and interpretation have been conducted exclusively by researchers. This raises the question of whether researchers might miss certain insights by not engaging patient-partners in data analysis and interpretation. The growth of POR has been accompanied with more patient-partner involvement in data analysis and interpretation.

Regarding data analysis, although it remains uncommon to engage patient-partners in quantitative data analysis, patient-partners appear to be increasingly involved in leading or supporting qualitative data analysis. For example, Stevenson (2014) engaged two youth with Down syndrome to analyze interview transcripts of other youth with Down syndrome describing their life goals [53]. For the analysis, a member of the research team prepared selections of interview transcripts, and the 2 patient-partners coded excerpts they considered “important” or “interesting.” The patient-partners conducted the coding over six 2-h sessions with a member of the research team. Through thematic analysis, the 2 patient-partners identified themes of friendship and connection as important. This example shows that patient-partners can not only support data analysis and interpretation, but also lead it. Stevenson empowered the youth partners, allowing them to identify themes most important to them and challenging assumptions about how patient-partners can contribute to analysis.

Engaging patient-partners in data analysis can lengthen the time required to conduct analyses, and training will likely need to be provided. Training can be provided by a member of the research team, as described by Stevenson. Alternatively, patient-partners could attend data analysis workshops that institutions provide to researchers and students.

Researchers can engage patient-partners in data interpretation by presenting data, including both qualitative and quantitative data, at group meetings and obtaining their perspectives and interpretations. Flicker (2006) reported that youth patient-partners often challenged the assumptions and interpretations made by researchers, and that this engagement added rigor to their interpretation [54]. Patient-partners can also help to add context to findings that researchers struggle to interpret. Lastly, when assisting with data interpretation, patient-partners can help to identify the story that exists in the data, the significance of the data, and form the foundation for knowledge mobilization materials.

### 4.3. Knowledge Mobilization

Traditionally, research findings have been presented to academic audiences by researchers. Here, we will discuss how patient-partners have joined researchers in delivering knowledge mobilization products to non-academic and academic audiences.

#### 4.3.1. Knowledge Mobilization to Non-Academic Audiences

Patient-partners have been involved in knowledge mobilization activities directed at the general public and patient population. For example, youth partners have been involved in making websites, writing newsletters, leading social media outputs, and making short videos [23]. The Canadian Premature Babies Foundation (CPBF), a foundation led by patient-partners, communicates new research evidence and knowledge to the lay public using blogs, infographics, social media campaigns, and videos [55]. The reach of the CPBF’s knowledge mobilization activities is wide; in 2023, their website had over 60,000 views in over 150 countries, and their YouTube channel had over 76,000 views [56]. Some keys to success for engaging patient-partners in knowledge mobilization activities include co-developing a comprehensive knowledge mobilization plan at the beginning of the project, allotting time for engagement, clarifying roles, and providing training and support as needed.

#### 4.3.2. Knowledge Mobilization to Academic Audiences

It has become increasingly common for patient-partners to co-deliver study findings in academic settings such as conferences. Nguyen et al. engaged youth and adult patient-partners in authoring conference abstracts, developing content for presentations and posters, and answering questions at a conference [57]. Co-delivering a presentation with a patient-partner in an academic setting allows for a natural way to embed the patient voice into the presentation, which can influence the audience in ways that cannot be achieved by a researcher presenting alone.

Peer-reviewed journal publications remain a key knowledge mobilization output in research. Engaging patient-partners in authorship can enhance trust between researchers and the patient community, and ensure that the patient voice is captured in the literature [58,59,60].

The growth of POR has sparked discussions about how to engage patient-partners in authorship and how to determine whether a patient-partner should be listed as an author or in the acknowledgments section. The International Committee of Medical Journal Editors (ICMJE) has developed criteria for determining authorship on publications. Richards et al. have provided a thoughtful commentary on how to apply ICMJE criteria to POR [61]. Patient-partners can be considered for authorship given the substantial role they play in study preparation and study execution activities. Patient-partners can be involved in drafting or revising the outline or any section of the manuscript. They might be particularly interested in drafting the plain language summary or a patient reflection in the discussion section. Patient-partners can also help to create figures and infographics to accompany the paper.

Ensuring that patient-partners are engaged early in the writing process will facilitate engagement; this should be addressed when discussing roles. Many patient-partners are unfamiliar with academic publication processes, so orienting them to topics and concepts such as author roles and publication terminology can mediate this challenge [61]. Lastly, it can take time for patient-partners to write and review manuscripts. It is important to adjust the timeline accordingly and find strategies to facilitate their work. For example, rather than sending patient-partner drafts to review independently, invite their feedback interactively at a meeting.

### 4.4. Peer Review

Peer review is a well-known stage of the research process whereby researchers assess whether a grant application should be funded based on the importance, logic, and necessity of the proposed work. As we transition to a research environment where patients have an equal choice in research, it has become increasingly apparent that patient-partners should also review grant applications [62].

For example, patient-partners can assess whether the research is worthwhile, whether the researchers have identified relevant outcome measures, if the patient-partner engagement plan is sufficient, and if the knowledge mobilization plan is appropriate. Granting organizations can tailor their peer review process in several ways to facilitate patient-partner peer review. Granting organization staff can provide customized review forms, extract relevant sections for the patient-partner to review, meet with the reviewer individually to clarify details in the grant, and engage them in a peer review panel. There appears to be limited peer-reviewed literature regarding how to engage patient-partners in peer review processes, making this an area for growth in the future.

## 5. Reporting and Evaluation in Patient-Oriented Research

### 5.1. Reporting and Evaluation of Project-Level Outcomes

As the application of POR continues to grow, so has the need to evaluate which engagement approaches and strategies work best. Several evaluation tools have been developed to measure different aspects of engagement from the perspectives of all team members [63]. Here, we describe evaluation tools that have been used in both adult and pediatric POR. The Public and Patient Evaluation Tool (PPEET) is a robust tool that can be used to evaluate the quality of engagement in research by assessing topics such as whether patient-partners have been oriented to their role, whether patient-partners are comfortable sharing their perspectives, whether patient-partners have an impact on the project, and whether patient-partners experience challenges working on the project [18,64].

The Community-Based Participatory Research (CBPR) questionnaire is a complementary tool to the PPEET [18]. The CBPR questionnaire evaluates topics such as the strength of the partnership between researchers and patient-partners, perceived levels of trust by researchers and patient-partners, and barriers and facilitators to engagement. Engagement experiences can also be captured through interviews. Although interviews are valuable, the challenges and barriers to engagement might be underreported when using interviews alone. It is important to ensure that challenges and failures are captured while evaluating engagement, as these are underreported in the literature [65]. Thus, evaluation tools that deliberately seek input about challenges and barriers are valuable because they identify areas for improvement.

In addition to evaluating the quality of engagement, it is important to have consistent and transparent reporting on the roles that patient-partners serve in a project. Reporting this information helps to establish a strong evidence-base for the field of POR. Currently, many authors either omit this information or report on it inconsistently throughout a paper. However, there are tools to help address this issue. The Guidance for Reporting Involvement of Patients and the Public version 2 (GRIPP2) reporting checklist prompts researchers to describe the roles of patient-partners using short narrative answers [66]. Tools such as the Involvement Matrix and subsections of the CBPR questionnaire can also be used to report on patient-partner roles. Additionally, certain journals, such as The BMJ, request that authors provide a paragraph detailing how patient-partners were involved in the project [67]. All these tools and strategies can help to improve our understanding of the breadth and depth of roles that patient-partners play in research.

### 5.2. Reporting and Evaluation of Systems-Level Outcomes

The tools mentioned above enable researchers to evaluate the benefits and impacts patient-partners have at a project-level. These tools do not measure whether benefits at the project level translate to systems-level benefits and impacts, such as whether POR results in improved health outcomes, a more patient-centric healthcare system, and a faster time to implement new interventions [68]. A recurring critique of POR is the lack of available evidence on whether POR results in these larger, systems-level results. Considering the additional effort and resources required to conduct POR, some researchers are hesitant to pursue POR in the absence of evidence supporting its effectiveness.

However, measuring systems-level changes is very complex, and attempting to draw a causal connection between a POR project and health outcomes is likely impractical given all the variables involved. Aubin et al. propose a framework for measuring the outcomes and impact of POR at various levels, including the project level, the research ecosystem level, and the health outcomes level, yet it remains unclear whether the latter can be measured [8]. While we hope that POR improves the quality of research and health outcomes, the notion that POR should only be pursued if these improvements can be proven detracts from the original moral, ethical, and political arguments to engage patients in research [65,69,70].

## 6. Conclusions

The POR movement continues to grow in pediatric populations. In this review, we describe the importance of building and maintaining rapport and respectful relationships with patient-partners. Active recruitment strategies are required to ensure that patient-partner populations are representative of the general population so that research evidence can be generated with all patients in mind. Clarifying, and then reviewing and revising, the roles of each patient-partner using tools such as the Involvement Matrix facilitates engagement at each stage of the research cycle and enhances communication between researchers and patient-partners. Patient-partners and researchers can build in time to reflect and identify strategies for their engagement throughout the research cycle. The literature cited in our review shows that engaging patient-partners in pediatric research is feasible, can add rigor to studies, and is feasible. We noted that reporting and evaluating is an area of growth, with more evaluation tools becoming available. Further research is needed to document methods and tools for evaluating engagement with youth and children in research.

In future research, it would be important to have detailed descriptions of participatory approaches that have been applied when engaging with children and youth. We observed that, compared to the adult literature, the pediatric literature does not contain many papers with thorough descriptions of the participatory approach that research uses. Many of the descriptive papers we highlighted in Section 4 were from the fields of participatory action research and community-based participatory research, and although these were useful for the purposes of this review, it is notable that this same level of detailed description is not seen in research funded by more recent POR programs from the NIHR, NIH, and CIHR. Thus, we identify increased reporting of the participatory approach as an area of growth for the field of pediatric POR.

## Figures and Tables

**Table 1:** Suggested strategies to involve patient-partners throughout the research cycle.

Research Stage		Activity	Examples of Strategies
How to begin POR	Pre-study activities	Building respectful relationships	Demonstrate interpersonal skills such as active listening, building trust, being open-minded, and being aware of body language.
			Participate in team trainings in conflict resolution and cultural sensitivity.
			Take time to explain the research context with partners.
		Recruiting patient-partners	Consider recruiting a diverse group of patient-partners.
			Network at events, including conferences, training sessions, and fundraisers.
			Use an online matching tool.
		Clarifying roles and establishing patient-partner committees	Use tools to have a conversation about the roles of research team members and patient-partners, such as terms of reference, patient-partner agreement, and the Involvement Matrix.
Engaging across the research cycle	Study preparation phase	Setting research priorities	Engage in priority-setting exercises, such as focus groups, surveys, workshops, and interviews. Consult resources from the James Lind Alliance to identify co-developed research priorities.
		Developing a research protocol and intervention	Involve patient-partners in identifying the research questions and research aims, informing the study design, selecting meaningful outcome measures, proposing appropriate questionnaires, determining inclusion and exclusion criteria for participants, and developing the recruitment strategy.
	Study execution phase	Participant recruitment	Involve patient-partners in recruitment, for example, by asking about recruiting through patient-only social media groups or in community settings.
		Data collection	Provide training to patient-partners on how to conduct and facilitate activities, such as interviews and focus groups.
			Orient patient-partners to the ethics of data collection, such as maintaining confidentiality, avoiding bias, and collecting data accurately.
			Support patient-partners in instances where participants recount stories that trigger traumatic memories from the patient-partners’ lived experience.
		Data analysis and interpretation	Present data, including qualitative and quantitative data, at group meetings for interpretations.
Knowledge Mobilization	KM products for academic and non-academic audiences	Co-presentations	Engage patient-partners in creating and delivering posters and talks. Guide patient-partners on developing materials and public speaking, if needed.
		Peer-reviewed publications	Engage with patient-partners early in the writing process and discuss roles for their involvement, including drafting or revising the manuscript.
			Provide co-authorship opportunities for patient-partners, aligning with the ICMJE criteria.

POR: patient-oriented research, KM: knowledge mobilization, ICMJE: International Committee of Medical Journal Editors.

## Data Availability

Not applicable.
